# Polo-like kinase 3 inhibits glucose metabolism in colorectal cancer by targeting HSP90/STAT3/HK2 signaling

**DOI:** 10.1186/s13046-019-1418-2

**Published:** 2019-10-26

**Authors:** Baochi Ou, Hongze Sun, Jingkun Zhao, Zhuoqing Xu, Yuan Liu, Hao Feng, Zhihai Peng

**Affiliations:** 1 Department of General Surgery, Shanghai General Hospital, Shanghai Jiao Tong University School of Medicine, No. 100, Haining Road, Shanghai, 200080 China; 2 Department of General Surgery, Ruijin Hospital, Shanghai Jiao Tong University School of Medicine, Shanghai, China

**Keywords:** Colorectal cancer, PLK3, Glucose metabolism, HSP90, STAT3, HK2

## Abstract

**Background:**

Polo-like kinase 3 (PLK3) has been documented as a tumor suppressor in several types of malignancies. However, the role of PLK3 in colorectal cancer (CRC) progression and glucose metabolism remains to be known.

**Methods:**

The expression of PLK3 in CRC tissues was determined by immunohistochemistry. Cells proliferation was examined by EdU, CCK-8 and in vivo analyses. Glucose metabolism was assessed by detecting lactate production, glucose uptake, mitochondrial respiration, extracellular acidification rate, oxygen consumption rate and ATP production. Chromatin immunoprecipitation, luciferase reporter assays and co-immunoprecipitation were performed to explore the signaling pathway. Specific targeting by miRNAs was determined by luciferase reporter assays and correlation with target protein expression.

**Results:**

PLK3 was significantly downregulated in CRC tissues and its low expression was correlated with worse prognosis of patients. In vitro and in vivo experiments revealed that PLK3 contributed to growth inhibition of CRC cells. Furthermore, we demonstrated that PLK3 impeded glucose metabolism via targeting Hexokinase 2 (HK2) expression. Mechanically, PLK3 bound to Heat shock protein 90 (HSP90) and facilitated its degradation, which led to a significant decrease of phosphorylated STAT3. The downregulation of p-STAT3 further suppressed the transcriptional activation of HK2. Moreover, our investigations showed that PLK3 was directly targeted by miR-106b at post-transcriptional level in CRC cells.

**Conclusion:**

This study suggests that PLK3 inhibits glucose metabolism by targeting HSP90/STAT3/HK2 signaling and PLK3 may serve as a potential therapeutic target in colorectal cancer.

## Background

Colorectal cancer (CRC) is one of the most common causes of tumor-related deaths worldwide with characteristics of unfavorable curative effect and poor prognosis [1]. Although progresses in screening and treatment have been made over the last decade, CRC remains a major health problem due to increasing morbidity and mortality [2]. Hence, elucidating the biologic characteristics and molecular mechanisms could be tremendously beneficial to the treatment of colorectal cancer.

To sustain continuous growth, cancer cells must reprogram canonical metabolic pathways to adapt to challenging hypoxic environments [3]. The Warburg effect, a core hallmark of cancer, is characterized by elevated glucose consumption and conversion of pyruvate to lactate even under normoxia [4]. Numerous evidences have revealed that activation of oncogenes or loss-of-function of tumor suppressors is responsible for aberrant metabolism in cancer cells [5]. For instance, the transcription factor c-Myc increases the expression of glycolytic genes, thereby promoting glycolysis and lactate secretion [6]. P53 helps cells adapt to limited periods of metabolic stress and resist the shift to glycolysis [7]. Although it is acknowledged that glycolysis occurs in CRC, the mechanism driving aerobic glycolysis remains largely unknown.

The Polo-like kinases (PLKs) are a family of highly conserved serine/threonine kinases, comprising five known members (PLK1–5). Each of them possesses a conserved N-terminal kinase domain and one or more polo-box domains (PBDs) at the C-terminus [8]. PLKs have been shown to regulate a number of important cellular processes, such as cycle progression, mitosis and DNA damage response [9]. Moreover, our previous investigation suggested that PLK2 promoted tumor growth and restrained cells apoptosis, while PLK3 seemed to have tumor-suppressive feature in CRC [10]. Recently, several studies have demonstrated that PLKs play critical roles in energy metabolism. Li et al. find that the phosphorylation of PTEN by PLK1 contributes to a tumor-promoting metabolic state [11]. In addition, targeting PLK1 significantly alters various genes associated with a decrease of cellular metabolism [12]. However, little is known about the overall pathophysiological contribution of PLK3 to CRC malignancy and glucose metabolism.

In this study, we found that low expression of PLK3 in CRC tissues was correlated with worse prognosis of patients and PLK3 inhibited cancer cells growth. We further demonstrated that PLK3 suppressed glucose metabolism through downregulating Hexokinase 2 (HK2) expression in CRC. Mechanically, PLK3 interacted with Heat shock protein 90 (HSP90) and contributed to its degradation, which led to a significant reduction of phosphorylated STAT3. The downregulation of p-STAT3 further depressed the transcriptional activation of HK2 in the context of PLK3/HSP90 signaling. These findings indicate that the PLK3/HSP90/STAT3/HK2 pathway serves as an important modulator of glucose metabolism in human colorectal cancer.

## Methods

### Cells and reagents

The human CRC cell lines and HEK293T were obtained from the American Type Culture Collection. SW480, SW620 and SW1116 are cultured in L-15 medium with 10% fetal bovine serum (FBS). HCT116, HT29 were maintained in McCoy’ 5A medium with 10% FBS, and CaCo2, RKO and DLD1 were cultured in RPMI-1640 medium with the same components. Cells were detected to be free of mycoplasma using a Mycoplasma Detection Kit (Roche, USA). MG-132 (Sigma) was used to inhibit proteasome-mediated proteolysis in some experiments.

### Immunohistochemistry (IHC)

The tissue microarray used in this study has been previously described [10]. Immunostaining was performed according to the manufacturer’s protocol (Immunostain SP kit, DakoCytomation, USA). The results of IHC were determined by the percentage of positive cells and staining intensity (staining intensity: negative = 0, weak = 1, moderate = 2, strong = 3; and percentage of cells stained: 0 = 0–1%, 1 = 1–5%, 2 = 6–29%, 3 = 30–59%, 4 = 60–100%). These two values were multiplied together to generate a single score for each case. All cases were grouped as either negative (score 0–3) or positive (score > 3).

### Quantitative real-time PCR (qPCR) and PCR array

Total RNA was extracted by using TRIzol reagent (Invitrogen). cDNA was synthesized with a TaKaRa PrimeScript RT reagent kit. The expression status of candidate genes and GAPDH were detected by using an ABI 7900HT Real-Time PCR system (Applied Biosystems). All of the reactions were done in triplicate. Primer sequences are shown in Additional file 1: Table S1. In addition, we performed the human glucose metabolism RT^2^ profiler PCR array, which contained 84 key genes involved in glycolysis, TCA cycle, Pentose Phosphate Pathway and so on.

### Immunoblotting

Immunoblotting was carried out as previously described [13]. Antibodies against PLK3 (4896), PCNA (2586), p-STAT3 (Tyr705, 9145), p-STAT3 (Ser727, 9134), p-p65 (3033) and total STAT3 (9139) were purchased from Cell Signaling Technology. Antibodies against HK2 (ab104836), HSP90 (ab13492), HIF-1α (ab2185), c-Myc (ab32072), MZF1 (ab64866) and GAPDH (ab9484) were purchased from Abcam.

### Stable transfection and transfection of oligonucleotides

Lentiviral pGLV-PLK3 particles and shRNA plasmids targeting PLK3 mRNA were purchased from Genepharma (Shanghai, China). Lentivirus particles were transfected into the CRC cells in the presence of polybrene and selected using 5 μg/ml puromycin. All stable transfected cells were tested regularly by immunoblotting to ensure the efficiency of upregulation or downregulation. miRNA mimics were synthesized by Ambion (Austin, USA). Small interfering RNAs (siRNAs) targeting HK2, STAT3 and HSP90 were synthesized by RiboBio (Guangzhou, China). Cells were transfected with the oligonucleotides using Lipofectamine 3000 (Invitrogen) and harvested 48 h post-transfection.

### CCK-8 and 5-ethynyl-2′-deoxyuridine (EdU) assay

CCK-8 proliferation assay was conducted as previously reported [14]. In addition, cells were incubated with EdU at a final concentration of 10 μM for 2 h and analyzed using the Click-iT EdU Assay (Roche, USA) according to the instructions. Images were captured under microscope and the percentage of EdU-positive cells was calculated.

### Glycolysis analysis and ATP production

Lactate Colorimetric Assay Kits (Biovision) and Glucose Uptake Colorimetric Assay Kits (Biovision) were used to examine the glycolysis process in cancer cells, according to the manufacturer’s instructions. The glycolytic capacity was examined by using the Glycolysis Stress Test Kit. ATP assay kit (Promega) was used to detect ATP production in tumor cells.

### Oxygen consumption rate (OCR) and extracellular acidification rate (ECAR)

ECAR and OCR were determined by the Bioscience XF96 Extracellular Flux Analyzer. Briefly, seeded onto 96-well plates and cultured overnight, cells were then washed with Seahorse buffer (Dulbecco’s modified Eagle’s medium with phenol red containing glucose, sodium pyruvate and glutamine). Later, 175 μL of Seahorse buffer plus 25 μL each of oligomycin, FCCP, and rotenone were automatically added to measure the OCR. The ECAR was calculated by injecting 25 μL each of glucose, oligomycin, and 2-deoxy-glucose (2-DG) into cells. The values of OCR and ECAR were normalized to the number of cells per well and are shown as the mean ± SD.

### Chromatin immunoprecipitation (CHIP)

ChIP assays were performed as previously described [15]. The chromatin immunoprecipitation Kit (Millipore) was used according to the manufacturer’s instructions. The presence of predicted transcription factor binding regions pulled by antibodies was examined by qPCR. Primers to detect gene promoter occupancy were shown in Additional file 1: Table S1.

### Luciferase reporter assay

The HK2 promoter was cloned into the pGL3-Basic luciferase plasmid (Promega, Madison, USA) to construct WT *P*_HK2_ reporter (Fig. 4d). A 9-bp sequence as the putative STAT3 binding site was deleted in the Mut *P*_HK2_ reporter (Fig. 4d) Tumor cells were then co-transfected with WT *P*_HK2_ or Mut *P*_HK2_ constructs. The primers were present in Additional file 1: Table S1. For PLK3 3′-UTR-Renilla luciferase reporter assay, each reporter construct was co-transfected into HEK293T cells together with luciferase plasmid pGL3 and miR-106b mimics or Control RNA. After 48 h of incubation, luciferase activities were measured using the Dual luciferase Reporter Assay System (Promega).

### Co-immunoprecipitation

Total cell lysates of cells were obtained in immunoprecipitation buffer, and then were incubated with anti-HSP90 antibody or the relative IgG control. The incubation was shaken on a rotating shaker for 2 h at 4 °C. Then, the proteins were immunoprecipitated by protein A/G Sepharose (Santa Cruz). Beads were collected and loading buffer was added to boil with them. The supernatants were then detected by immunoblotting.

### Xenograft model

Forty 4-week-old male BALB/c nude mice were randomly divided into 4 groups (10 for each group). One million tumor cells in 100 μl PBS were injected subcutaneously. Tumor sizes were measured every 6 days and calculated as: V = (Width^2^ × Length)/2. Xenografts were collected at 36th day for protein extraction. All experiments were conducted with the approval and guidance of the Animal Ethics Committee (Shanghai Jiao Tong University School of Medicine).

### Statistical analysis

The data are presented as the means ± SD of at least three independent experiments. Two-tailed unpaired Student *t* tests and one-way analysis of variance were used for the data analysis. Kaplan-Meier method was used to assess patients’ survival outcome. Differences were considered significant at *, *P* < 0.05; **, *P* < 0.01; and ***, *P* < 0.001.

## Results

### Clinical significance of PLK3 in colorectal cancer

**Fig. 1:**
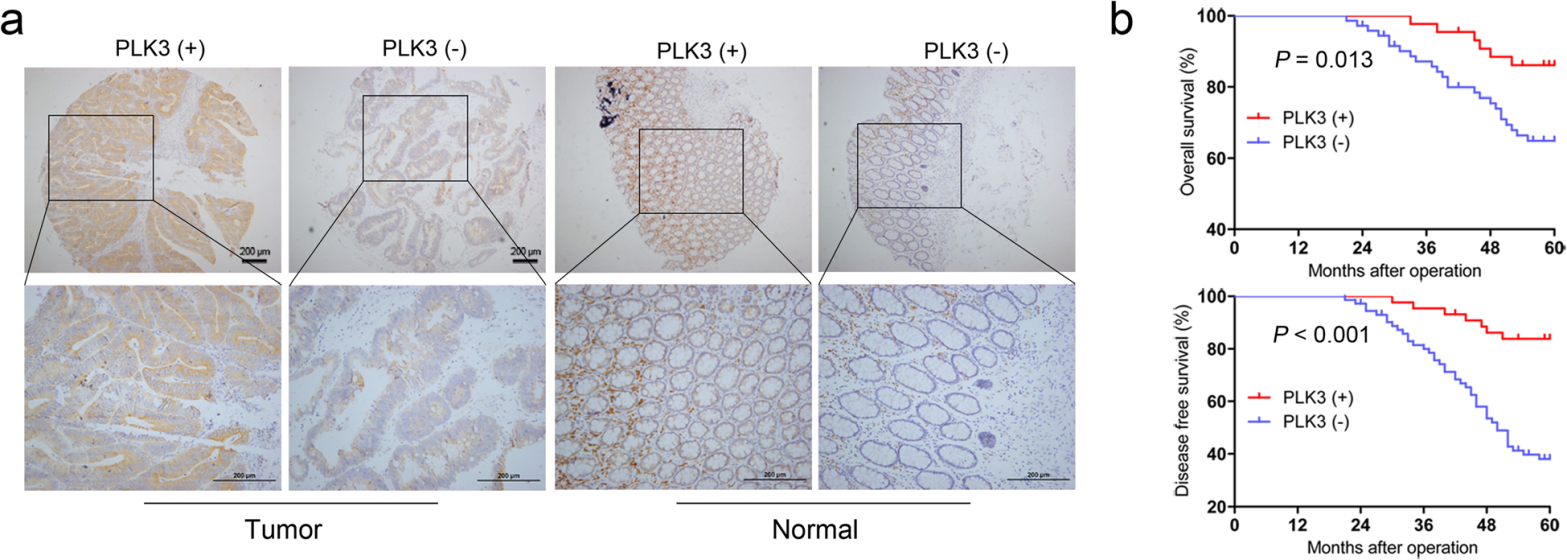
Clinical significance of PLK3 in CRC patients. **a** PLK3 expression level in tumor tissues and the paired normal tissues was evaluated by IHC in the tissue microarray. **b** CRC patients with negative expression of PLK3 presented worse overall survival, and disease-free survival compared with that of positive expression of PLK3

Our previous research has showed that PLK3 is lowly expressed in CRC tissues relative to matched normal tissues [10]. In this study, we further analyzed PLK3 expression in a tissue microarray containing 116 pairs of cancerous and matched normal tissue by IHC (Fig. 1a). Moreover, the correlation between pathologic factors and PLK3 expression were compared. In these cases, the negative expression of PLK3 was detected in 72 (62.1%) of the tumor tissues, whereas 49 (42.2%) of the adjacent normal specimens showed a negative signal (Table 1). Furthermore, a significant association was observed between the PLK3 negative group and positive group in tumor size (*P* = 0.008), lymphatic metastasis (*P* = 0.027) and TNM stage (*P* = 0.019) (Table 1). Kaplan–Meier analysis revealed that patients in the PLK3^negative^ group had a significantly poorer overall survival than those in the PLK3^positive^ group (*P* = 0.013; Fig. 1b). In addition, PLK3^negative^ patients had a shorter disease-free survival (*P* < 0.001; Fig. 1c). However, multivariate analyses indicated that PLK3 expression was not an independent prognostic factor for survival in CRC patients (data not shown).

**Table 1:** Relationship between PLK3 expression and clinicopathologic variables in 116 CRC patients

Variable	Case (*n* = 116)	PLK3 expression		*P* value
		Negative	Positive	
Tissues				0.003
Normal tisssues	116	49	67	
Carcinoma	116	72	44	
Gender				0.936
Male	68	42	26	
Female	48	30	18	
Age				0.623
≤ 65	52	31	21	
>65	64	41	23	
Location				0.301
Left hemicolon	12	10	2	
Right hemicolon	33	22	11	
Sigmoid colon	24	14	10	
Rectum	47	26	21	
Tumor size (cm)				0.008
≤ 4 × 3	53	26	27	
> 4 × 3	63	46	17	
Tumor histology				
Tubular	98	60	38	0.796
Mucinous	16	11	5	
Papillary	2	1	1	
Extent of invasion				0.332
T1+ T2	31	17	14	
T3+ T4	85	55	30	
Lymphatic metastasis				0.027
N0	56	29	27	
N1 + N2	60	43	17	
Metastasis				0.444
M0	105	64	41	
M1	11	8	3	
TNM stage				0.019
I + II	55	28	27	
III + IV	61	44	17	
CEA level				
< 5.0	89	54	35	0.574
≥ 5.0	27	18	9	

### PLK3 inhibits proliferation and glucose metabolism of CRC cells

**Fig. 2:**
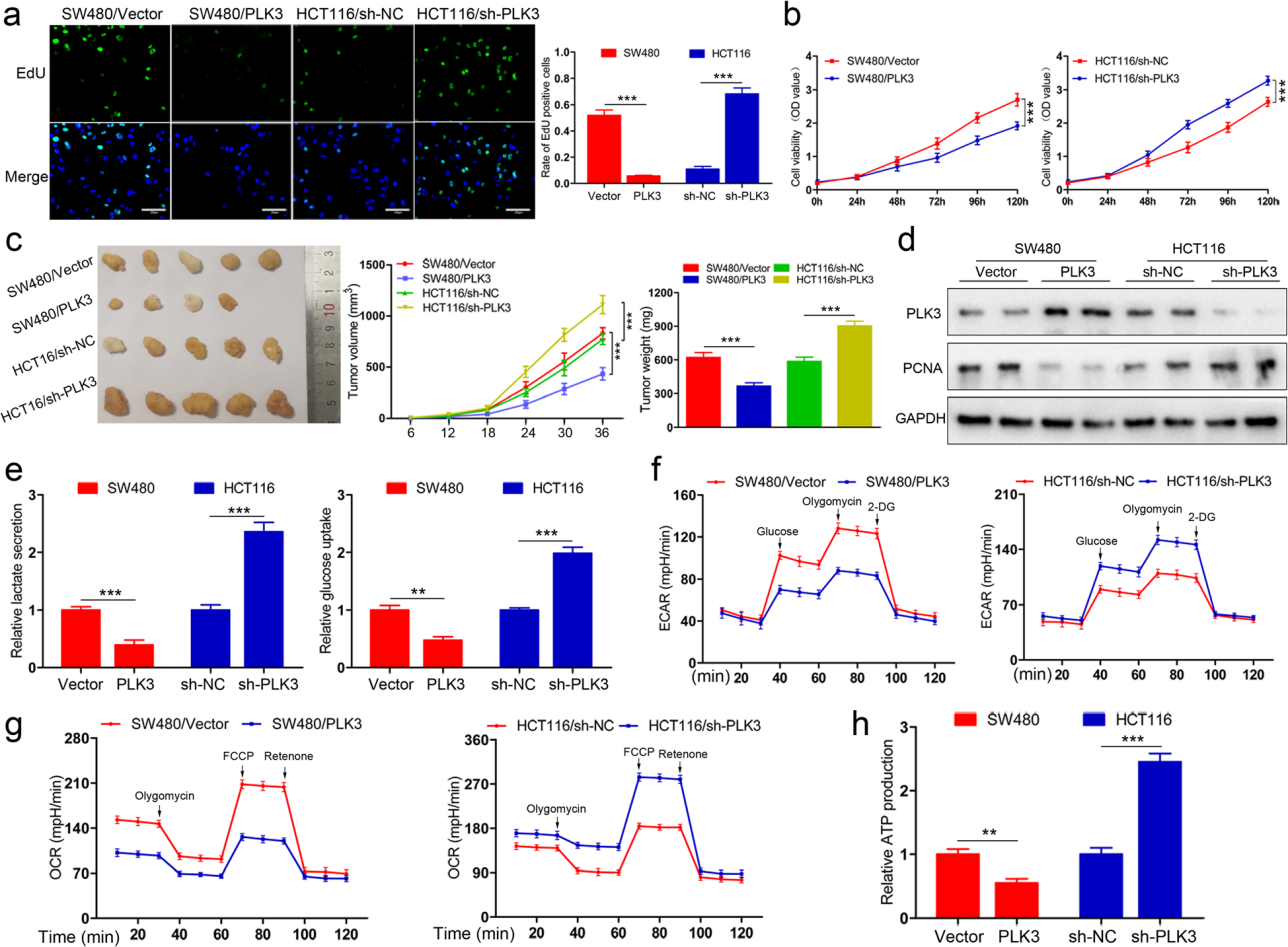
PLK3 suppresses proliferation and glucose metabolism of CRC cells. **a** Cell proliferation as measured by EdU assay was inhibited by PLK3 overexpression in SW480 cells and promoted by PLK3 knockdown in HCT116 cells. Scar bars, 200 μm. **b** CCK-8 assay showing PLK3 overexpression reduces cells viability of SW480 and PLK3 depletion enhances the viability of HCT116 cells. **c** Morphological observation of formed xenografts, the volumes measured every 6 days and average weight of tumors. **d** Immunoblotting analysis of PLK3 and PCNA in tumor samples. **e** Lactate production and glucose consumption of SW480 and HCT116 cells were determined. **f** Analysis of ECAR of PLK3-overexpressing SW480 cells and HCT116 cells with PLK3 knockdown. **g** Analysis of OCR of PLK3-overexpressing SW480 cells and HCT116 cells with PLK3 knockdown. **h** ATP production was examined in SW480 and HCT116 cells. Data represent the mean ± SD of at least three independent experiments. **P < 0.01, ***P < 0.001

The expression of PLK3 in eight CRC cell lines was detected. As shown in Additional file 2: Figure S1a and b, PLK3 was differently expressed and especially high in HCT116 and HT29, but low in SW480 and RKO. Thus, we determined to knock down PLK3 in HCT116 cells, and selected SW480 for exogenous PLK3 overexpression. The efficiency of transfection was confirmed by immunoblotting (Additional file 2: Figure S1c). PLK3 overexpression or silencing exert no influence on the expression of other PLK members (Additional file 2: Figure S1d). We then performed EdU incorporation to examine the effect of altering PLK3 levels on cells proliferation. As shown in Fig. 2a, PLK3 overexpression suppressed proliferative ability of SW480 cells and PLK3 knockdown promoted HCT116 cells proliferation. In addition, CCK-8 assay also confirmed the effect of PLK3 on proliferation of CRC cells (Fig. 2b). To study the effects of PLK3 in vivo, we subcutaneously injected nude mice with tumor cells. As expected, PLK3 overexpression inhibited tumor growth and reduced tumor weight in the xenograft mouse model, whereas PLK3 knockdown had the opposite effects (Fig. 2c). Subsequent immunoblotting analysis demonstrated that PCNA (marker of proliferation) expression was significantly influenced by PLK3 in tissues from xenograft tumors (Fig. 2d).

To determine whether cell proliferation mediated by PLK3 reflects a change in glucose metabolism, we first examined lactate production and glucose uptake, two primary indicators of the Warburg effect in tumor cells. As shown in Fig. 2e, ectopic expression of PLK3 reduced lactate production and glucose uptake of SW480 cells, while silencing PLK3 increased lactate production and glucose uptake of HCT116 cells. We then detected the ECAR, which reflects the lactate–induced acidification of the medium surrounding cells. The data indicated that the ECAR was increased in PLK3-silencing cells, whereas PLK3 overexpression decreased the ECAR value and may play a suppressive role in lactate formation (Fig. 2f). Additionally, OCR, the indicator of mitochondrial respiratory capacity, was measured in HCT116 and SW480 cells. Interestingly, PLK3-overexpressing cells exhibited lower OCR relative to the control. Conversely, the OCR was increased in PLK3-depleted HCT116 cells (Fig. 2g). ATP is indispensable to rapid proliferation and metastasis of cancer cell. Herein, we found that PLK3 overexpression exerted an inhibitory effect on ATP production, while PLK3 knockdown increased ATP levels in HCT116 cells (Fig. 2h). Altogether, these findings suggest PLK3 plays a critical role in proliferation and glucose metabolism of CRC cells.

### HK2 is responsible for PLK3-mediated CRC cells glycometabolism

**Fig. 3:**
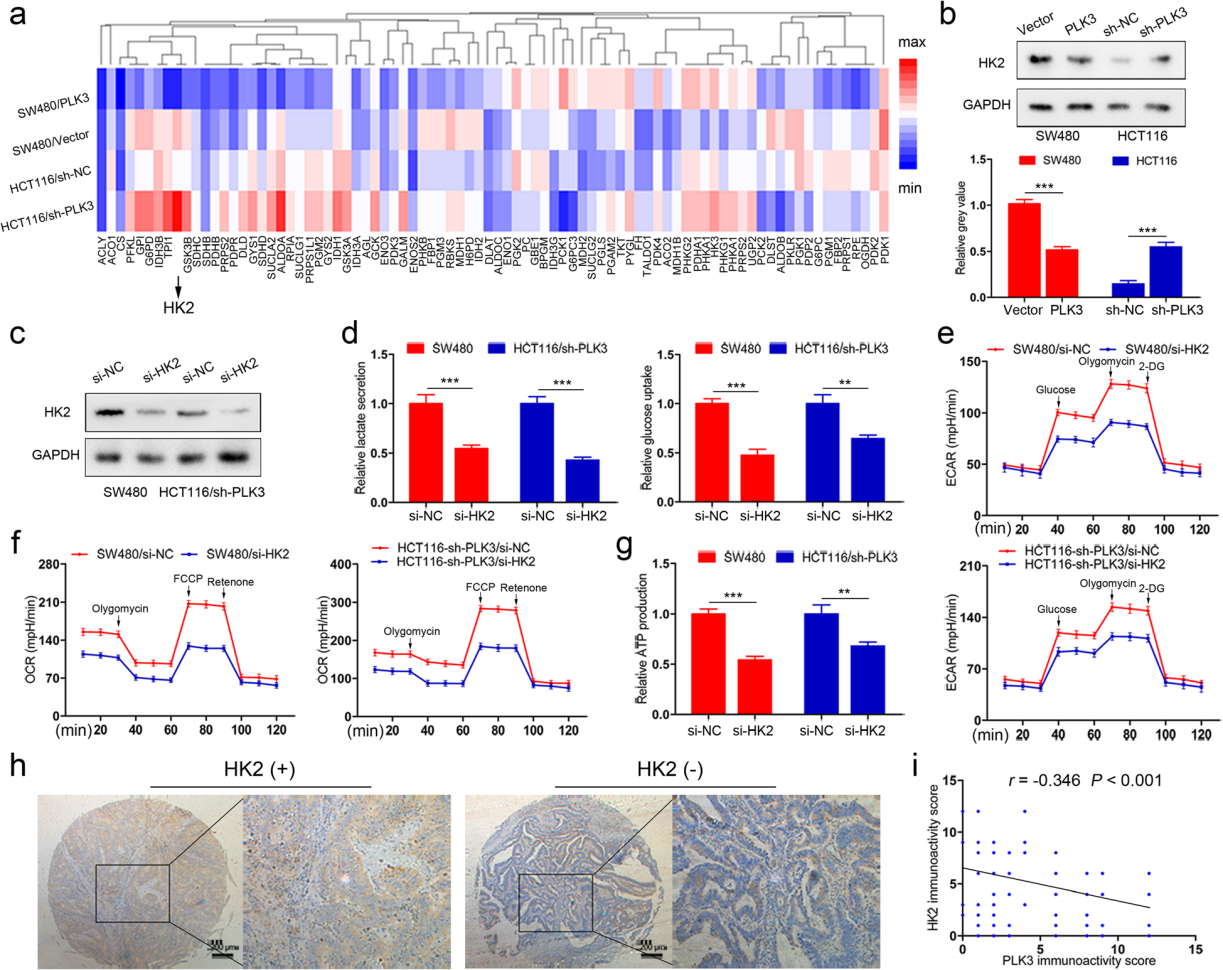
HK2 plays a role in PLK3-mediated CRC cells glycometabolism. **a** A total of 84 genes are quantified using a human glucose metabolism PCR Array. The color scheme represents gene expression changes on a log2 scale. **b** Cells transfected with pGLV-PLK3 and sh-PLK3, respectively, were subject to immunoblotting. **c** Immunoblotting analysis of HK2 in SW480 and HCT116/sh-PLK3 cells with HK2 depletion. **d** Lactate production and glucose consumption were determined in SW480 and HCT116/sh-PLK3 cells with HK2 knockdown. **e** Analysis of ECAR of SW480 and HCT116/sh-PLK3 cells with HK2 inhibition. **f** Analysis of OCR of SW480 and HCT116/sh-PLK3 cells with HK2 inhibition. **g** ATP production was examined in SW480 and HCT116/sh-PLK3 cells with HK2 knockdown. **h** Representative images of HK2 positive and negative staining in the tissue microarray. **i** Expression correlation of PLK3 and HK2 was analyzed in 116 CRC patients using IHC. Data represent the mean ± SD of at least three independent experiments. *P < 0.05, **P < 0.01, ***P < 0.001

To elucidate the molecular mechanisms for PLK3-mediated glycometabolism, we analyzed differentially expressed genes for cancer cells using a human glucose metabolism PCR array. Among 84 genes examined, several genes were significantly upregulated in cells with low expression of PLK3 (SW480, HCT116/sh-PLK3) relative to their controls (Fig. 3a). HK2, an enzyme that catalyzes the first and irreversible step of glycolysis, was selected for further investigation because it was the most upregulated gene. Indeed, immunoblotting analysis confirmed that PLK3 overexpression reduced HK2 protein expression in SW480 and that PLK3 knockdown improved the levels of this protein in HCT116 cells (Fig. 3b). To further explore whether HK2 is crucial for PLK3-mediated cell glycolysis, siRNAs were used to knockdown HK2 expression in cells with low expression of PLK3 (SW480 and HCT116/sh-PLK3), whose effects were confirmed by immunoblotting (Fig. 3c and Additional file 3: Figure S2a). After si-HK2 treatment, the increased glucose uptake and lactate production induced by PLK3 silencing were dramatically abolished in tumor cells (Fig. 3d). Similarly, the values of ECAR (Fig. 3e) and OCR (Fig. 3f) were decreased remarkably after HK2 silencing in tumor cells. ATP production was also suppressed upon HK2 knockdown in SW480 and HCT116/sh-PLK3 cells (Fig. 3g).

We then analyzed HK2 expression in the same tissue microarray (Representative images: Fig. 3h). As expected, HK2 was highly expressed in tumor samples compared to normal samples (67.2% versus 44.8%, *P* = 0.001, Additional file 1: Table S2). Furthermore, our results showed a negative association between PLK3 and HK2 expression (Pearson’s correlation, *r* = − 0.346, *P* < 0.001, Fig. 3i) in CRC tissues. These data indicate that PLK3 plays a role in CRC glucose metabolism by regulating HK2 expression.

### PLK3-mediated STAT3 transcriptionally regulates HK2 expression in CRC cells

**Fig. 4:**
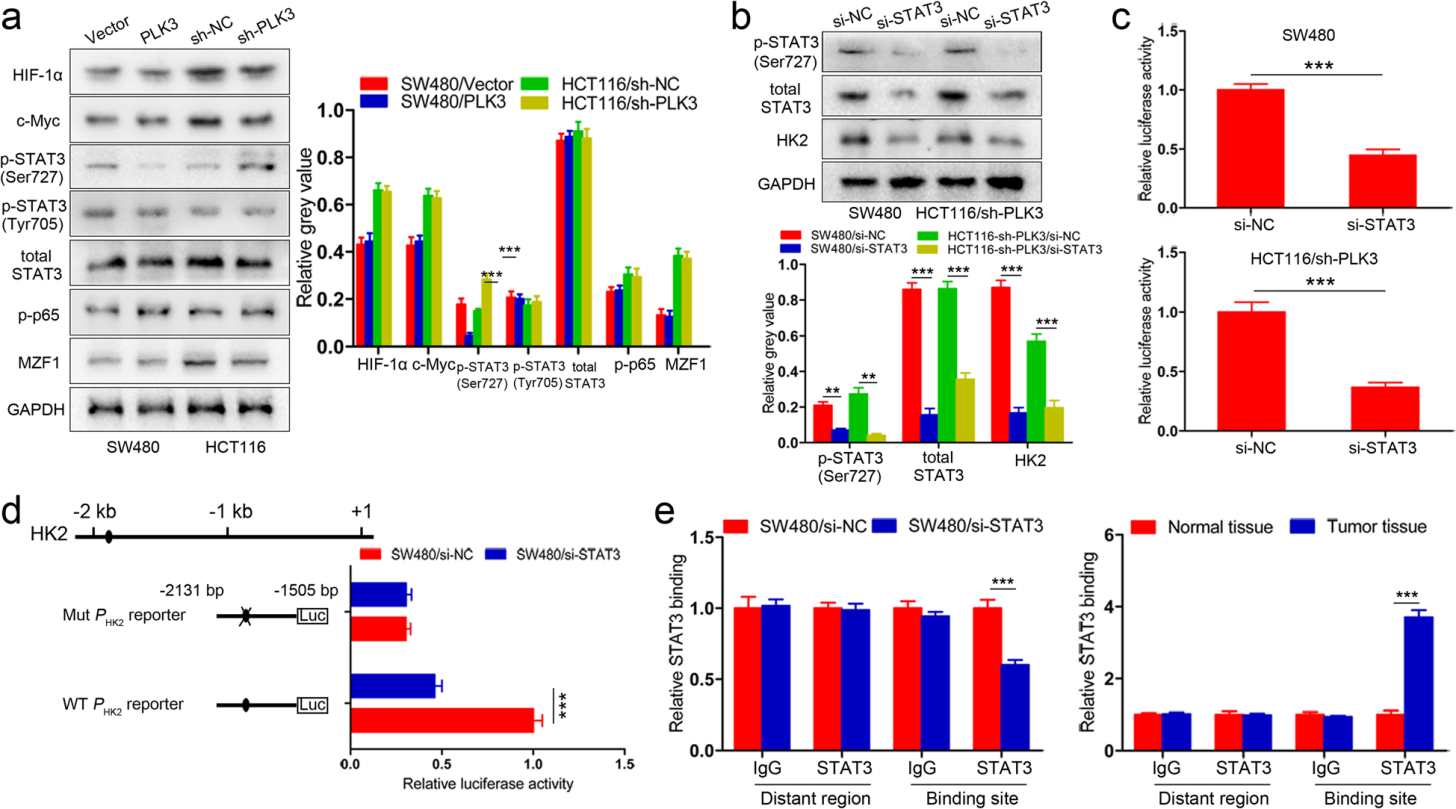
STAT3 is involved in the regulation of HK2 by PLK3 in CRC cells. **a** Immunoblotting analysis of HIF-1α, c-Myc, p-STAT3 (Ser727), p-STAT3 (Tyr705), total STAT3, p-p65 and MZF1 in PLK3-overexpressing SW480 cells and PLK3-depleted HCT116 cells. **b** Immunoblotting analysis of p-STAT3 (Ser727), total STAT3 and HK2 in SW480 and HCT116/sh-PLK3 cells with STAT3 knockdown. **c** The HK2 promoter luciferase construct (− 2131/− 1505) HK2 was co-transfected with si-STAT3, and promoter activities were detected. **d** WT or Mutated HK2 promoter constructs were co-transfected with si-STAT3, and luciferase activities were determined. The schematic constructs are shown (left), and the bar graphs present the relative luciferase activity in each sample (right). **e** ChIP assays of STAT3 occupancy on the HK2 promoter. The level of STAT3 binding site in SW480/si-NC cells and normal tissues was set to 1.0, and that in others was normalized relative to the control. Data represent the mean ± SD of at least three independent experiments. **P < 0.01, ***P < 0.001

To understand how PLK3 suppresses HK2 gene expression in CRC cells, we hypothesized that HK2 transcription was mediated by one or more transcription factors chaperoned by PLK3. We performed a literature search and shortlisted five proteins that have been reported to regulate HK2 transcription in human cells, namely, HIF-1α, c-Myc, STAT3, NF-κB, Myeloid Zinc Finger-1 (MZF1) [16–20]. The protein levels of these transcription factors were then examined. As indicated in Fig. 4a, ectopic expression of PLK3 led to downregulation of p-STAT3 (Ser727), while silencing PLK3 dramatically activated p-STAT3 (Ser727) expression. The expression of other proteins (Fig. 4a) exhibited no significant changes. It is known that the transcriptional activation of STAT3 depends on its phosphorylation at tyrosine 705 or serine 727 residue, which leads to different functions [21]. Indeed, when using siRNAs to knockdown p-STAT3 (Ser727) in SW480 and HCT116/sh-PLK3 cells, we confirmed that blockade of p-STAT3 (Ser727) significantly suppressed HK2 expression levels (Fig. 4b).

Next, we used TransFac and Genomatix software [22] to analyze the upstream sequences of HK2, ranging from − 2000 to + 200 bp relative to the transcription start site. Only one putative binding site was identified within the human HK2 promoter region. We then constructed luciferase reporters under control of either the wild-type HK2 promoter or a mutant promoterwith a deletion of the 9-bp STAT3 binding site (named as WT *P*_HK2_ reporter or Mut *P*_HK2_ reporter, respectively). The results demonstrated that this site was critical for STAT3-induced HK2 activation (Fig. 4c and d). Subsequent CHIP assays also verified the direct binding of STAT3 to the predicted site of HK2 promoter in CRC cells and tissues (Fig. 4e). Altogether, these findings suggest that STAT3 is a direct transcriptional activator for HK2 in human colorectal cancer.

### HSP90 stabilization is involved in PLK3-mediated CRC glucose metabolism

**Fig. 5:**
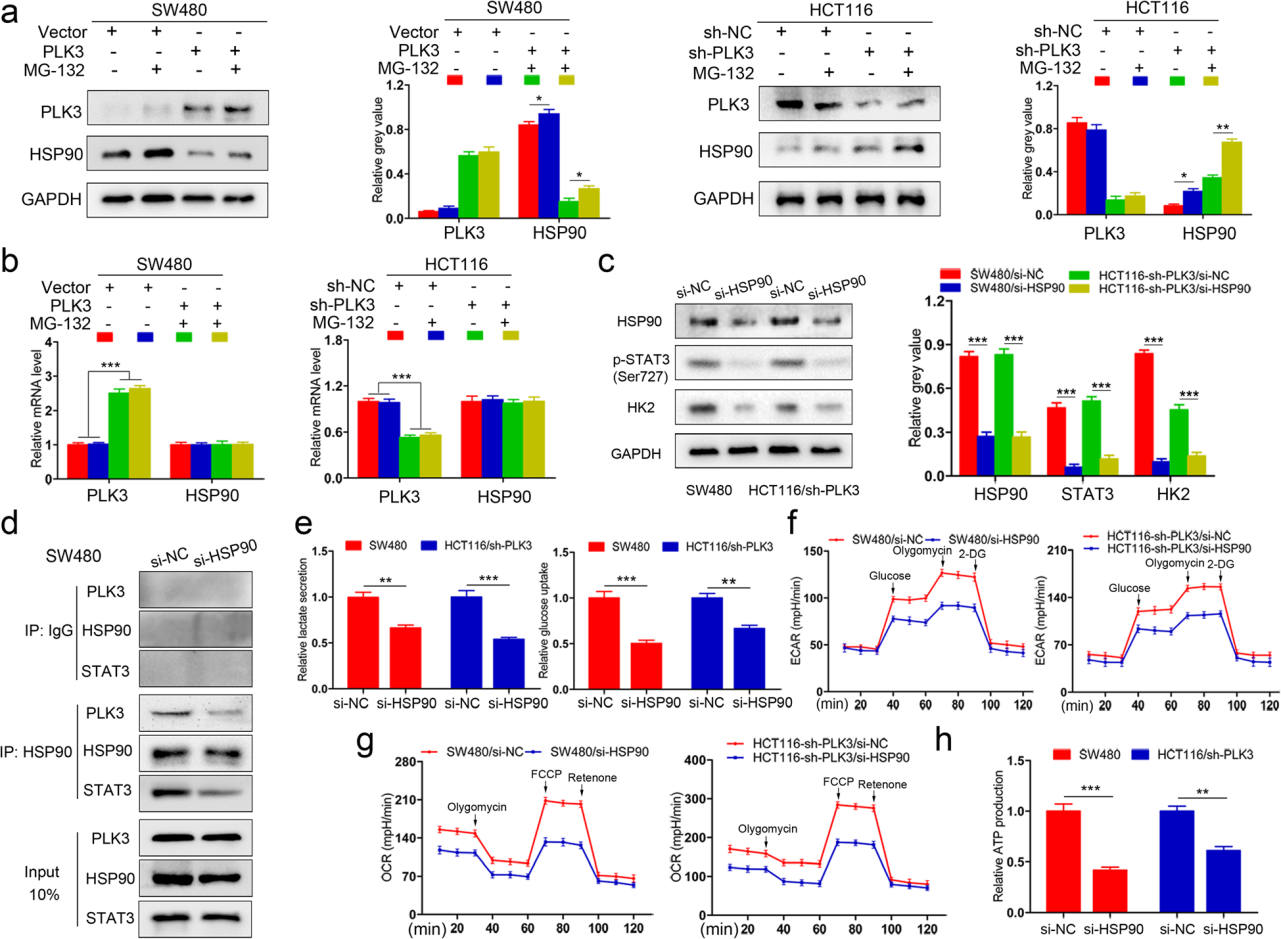
PLK3-medatied HSP90 plays a crucial role in glucose metabolism of CRC cells. **a** SW480 and HCT116 cells transfected with pGLV-PLK3 and sh-PLK3, respectively, were determined by immunoblotting for PLK3 and HSP90. MG-132 (5 μg/ml) was used to treat cells for 4 h before harvesting. Densitometry represents the expression of the proteins relative to GAPDH. **b** PLK3 overexpression or knockdown did not change HSP90 mRNA expression levels, detected by qPCR. **c** Immunoblotting analysis of HSP90, p-STAT3 (Ser727) and HK2 in SW480 and HCT116/sh-PLK3 cells with HSP90 silencing. **d** HSP90 was co-immunoprecipitated with PLK3 and STAT3 in SW480 cells with and without HSP90 depletion. **e** Lactate production and glucose consumption were determined in SW480 and HCT116/sh-PLK3 cells with HSP90 knockdown. **f** Analysis of ECAR of SW480 and HCT116/sh-PLK3 cells with HSP90 inhibition. **g** Analysis of OCR of SW480 and HCT116/sh-PLK3 cells with HSP90 inhibition. **h** ATP production was examined in SW480 and HCT116/sh-PLK3 cells with HSP90 knockdown. Data represent the mean ± SD of at least three independent experiments. *P < 0.05, **P < 0.01, ***P < 0.001

Since the direct interaction of PLK3 and STAT3 was not observed in CRC cells (data not shown), the mechanism that bridges these two molecules was further explored. Previous researches have demonstrated that PLK3 may play a role in oncogenesis by mediating various substrates, such as PTEN, HIF-1α, HSP90 and so on [23–25]. In this study, we screened the expression of these substrates and found that HSP90 was significantly influenced by PLK3 overexpression or silencing (Fig. 5a), whereas HSP90 mRNA levels varied slightly (Fig. 5b). The stability of HSP90 could be rescued by MG-132 treatment, suggesting the regulation between PLK3 and HSP90 involves phosphorylation-mediated degradation by proteasome (Fig. 5a). Moreover, when using siRNAs to knockdown HSP90 expression in the cells with low expression of PLK3, we found that si-HSP90 significantly suppressed the expression of p-STAT3 (Ser727) and HK2 (Fig. 5c). In addition, co-immunoprecipitation assays showed that both PLK3 and STAT3 were detected in the immunoprecipitatation products (Fig. 5d and Additional file 3: Figure S2b). This confirmed the interactions between PLK3, STAT3 and HSP90, which were significantly inhibited when cells were transfected with si-HSP90 (Fig. 5d and Additional file 3: Figure S2b). Subsequent experiments demonstrated that silencing HSP90 impeded lactate production, glucose uptake, mitochondrial respiration, ECAR, OCR and ATP production in SW480 and HCT116/sh-PLK3 cells (Fig. 5e-h). Thus, our data indicate that PLK3-medatied HSP90 plays a critical role in glucose metabolism of CRC cells.

### PLK3 expression is directly suppressed by miR-106b in CRC cells

**Fig. 6:**
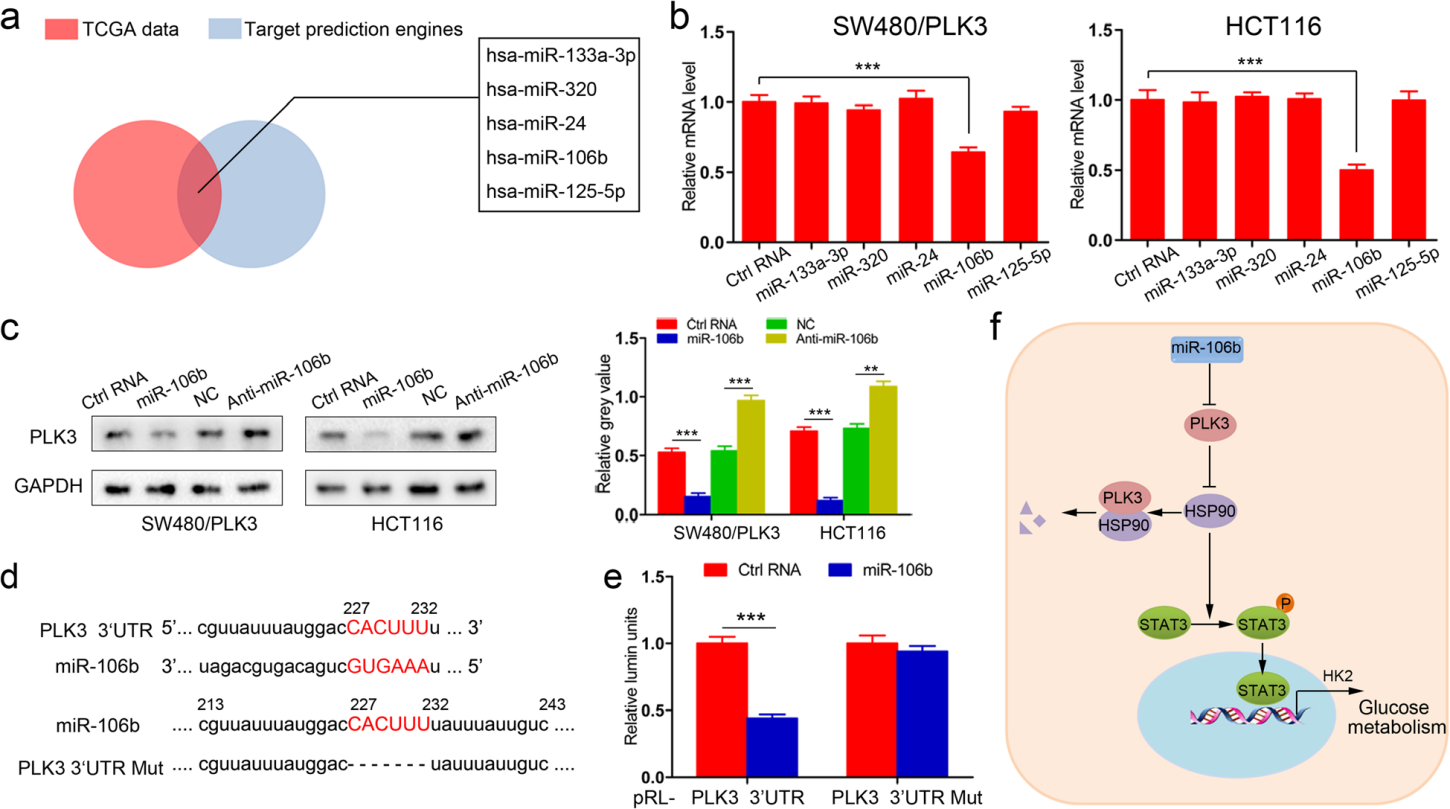
miR-106b inhibits PLK3 expression by targeting the 3′-UTR of PLK3 mRNA. **a** A schematic diagram of the protocol used to search for miRNAs that target PLK3. **b** miR-106b mimics significantly reduced PLK3 mRNA expression, determined by qPCR. **c** Immunoblotting analysis of PLK3 in SW480/PLK3 and HCT116 cells transfected with miR-106b or anti-miR-106b. Densitometry represents the expression of the protein relative to GAPDH. **d** TOP, predicted miR-106b binding site in the 3′-UTR of PLK3 mRNA; bottom, sequences of wild-type and mutated PLK3 3′-UTR-Renilla luciferase reporters. **e** Relative activity of luciferase reporters with PLK3 3′-UTR after co-transfection with miR-106b mimics in 293 T cells. **f** Model of PLK3 as a key regulator that orchestrates glucose metabolism in colorectal cancer through the HSP90/STAT3/HK2 signaling. Data represent the mean ± SD of at least three independent experiments. **P < 0.01, ***P < 0.001

MicroRNAs (miRs) are small, noncoding RNAs that coordinate the gene expression at the post-transcriptional level [26]. To understand the mechanism responsible for the low expression of PLK3 in CRC, we hypothesized that PLK3 was directly regulated by microRNAs. The Cancer Genome Atlas (TCGA) was screened for miRs that were significantly dysregulated in CRC (fold change ≥2 or ≤ 0.5). Candidate targets were searched using the prediction tools including miRanda, miRWalk and TargetScan. 5 microRNAs were finally identified and selected for further investigation (Fig. 6a). Interestingly, only miR-106b mimics-transfected cells exhibited a significant decrease in the mRNA expression of PLK3 (Fig. 6b). Indeed, ectopic expression of miR-106b decreased PLK3 protein levels in cells with high PLK3 expression (SW480/PLK3 and HCT116), whereas inhibition of miR-106b by anti-miR-106b led to a converse effect (Fig. 6c).

To confirm the regulation of PLK3 by miR-106b, we constructed the luciferase reporters containing the wild-type (WT) or mutated 3′-UTR sequences of PLK3 (Fig. 6d). The reporters were co-transfected with miR-106b mimics or negative control RNAs into HEK293T cells. Interestingly, we observed that the luciferase activity of wild-type vector, but not its mutant counterpart, was significantly suppressed by miR-106b (Fig. 6e). Collectively, the schematic diagram of our study is shown in Fig. 6f.

## Discussion

In recent decades, the Warburg effect has been highlighted as a hallmark of cancer [27]. Even in the presence of an oxygen supply, tumor cells preferentially utilize glycolysis rather than mitochondrial oxidative phosphorylation to produce ATP for biosynthesis. The reprogramming of tumor metabolism is suggested to be controlled by various oncogenic signals [28, 29]. In colorectal cancer, the pro-inflammatory cytokines TNFα and IL-17 have been reported to cooperatively stimulate glycolysis [30]. The study by Tambe et al. suggests the tumor suppressor drs inhibits Warburg effect via lactate dehydrogenase-B [31]. Furthermore, Che-1 mediates HIF-1α stabilization to affect glucose metabolism in the response to hypoxia [32].

PLKs are dysregulated in many types of cancer, such as liver cancer, brain cancer and ovarian cancer [9]. To date, only PLK1 was identified as an important coordinator of glucose metabolism in tumor cells [11, 12]. The roles of PLK2–5 in cancer glucose metabolism remain to be elucidated. Herein, we found that PLK3 was lowly expressed in 62.1% of tumor samples examined, suggesting that alteration of PLK3 level is a frequent event in human CRC. Moreover, a remarkable correlation between PLK3 expression and tumor size, lymphatic metastasis and TNM stage was found. PLK3 negative expression was significantly associated with poor prognosis of CRC patients. Subsequent in vitro functional assays demonstrated that PLK3 inhibited CRC cells proliferation. In vivo experiments also supported a tumor-suppressive function of PLK3 in CRC. Thus, our investigations focused on the role and molecular mechanism of PLK3 in CRC glucose metabolism.

Next, a series of aerobic glycolysis-related assays were performed. We found that overexpression of PLK3 dramatically inhibited glycolysis, whereas silencing PLK3 promoted glucose metabolism in CRC cells. To explore the potential mechanism, we profiled the differentially expressed genes involved in glycolysis, tricarboxylic acid cycle, pentose phosphate pathway and so on. HK2 was identified as a candidate target and chosen for further exploration. HK2, the major isozyme of hexokinase, catalyzes the first irreversible step in the glycolytic pathway by phosphorylating glucose to G6P [33]. It has been reported that HK2 is exclusively upregulated in many types of cancers and plays a key role in the Warburg effect [34]. In this study, HK2 expression level was found to be decreased under condition of PLK3 overexpression, while silencing PLK3 increased HK2 expression in tumor cells. Inhibition of HK2 significantly suppressed glucose metabolism of PLK3-depleted CRC cells. Furthermore, PLK3 was inversely associated with HK2 protein expression in CRC tissues. These data provide clues on the mechanism that PLK3 might impede CRC glycolysis by targeting HK2.

We then explored the potential signal pathways implicated in the regulation of HK2 expression. A comprehensive literature search was performed and we shortlisted five transcription factors that have been reported to regulate HK2 transcription in human cells. The results showed that p-STAT3 (Ser727) expression was significantly inhibited by PLK3 overexpression, while PLK3 depletion increased p-STAT3 (Ser727) protein levels. Moreover, CHIP and luciferase reporter assays demonstrated that STAT3 directly bound to the promoter region of HK2 and contributed to its expression. Recently, STAT3 signaling has been shown to reprogram cellular metabolism in human malignancies. The study by Lin et al. [35] indicates that palmitic acid limits glycometabolism through the attenuation of STAT3 pathway in hepatocellular carcinoma. Furthermore, STAT3 signaling participates in the pyruvate kinase isoenzyme type M2-mediated glycolysis of breast cancer cells [36]. Our data herein suggested STAT3 played a crucial role in HK2 expression and glucose metabolism of human CRC cells. However, we could not exclude the possibility that other forms of STAT3 phosphorylation may be involved in HK2 downregulation.

It has been recognized that the PBDs located in the C-terminal region of PLKs are responsible for their functions. The PBD acts as a key mediator via phosphorylation-dependent interactions with downstream targets [8]. Previous researches have revealed that PLK3 plays a role in carcinogenesis by regulating various substrates [37]. In this study, we screened the expression of these targets and found that HSP90 was significantly influenced by PLK3 silencing or overexpression. The regulation of HSP90 by PLK3 involves proteasome-mediated degradation. Moreover, using siRNAs to target HSP90, we observed that the expression levels of p-STAT3 (Ser727) and HK2 were significantly downregulated. In addition, the interactions between PLK3, STAT3 and HSP90 were validated by co-immunoprecipitation in CRC cells. These findings suggest PLK3 directly binds to HSP90 and contributes to the following degradation of HSP90 and inactivation of STAT3. Subsequent experiments further showed that HSP90 inhibition suppressed glucose metabolism in the PLK3-depleted cells. Altogether, our investigations demonstrate that PLK3/HSP90/STAT3/HK2 signaling plays a critical role in glycometabolism of CRC cells.

Emerging evidence indicates that microRNAs have important functions during tumor progression through posttranscriptional regulation of gene expression [26]. Furthermore, microRNAs have also been documented as crucial mediators in the reprogramming of cellular energy metabolism [38]. In this study, we combined the data from TCGA as well as microRNAs prediction engines and identified miR-106b as the upstream regulator of PLK3 in CRC cells. Luciferase reporter assays also confirmed that PLK3 was a direct target of miR-106b in HEK293T cells. Previously, miR-106b has been reported as a contributor for CRC cell migration and invasion [39]. Moreover, miR-106b could enhance CRC cell radioresistance and tumor-initiating capacity [40]. Our results herein suggested miR-106b may have a function through targeting PLK3 in CRC cells. However, the role of miR-106b in cancer glucose metabolism remains to be clarified.

## Conclusions

In conclusion, the present study demonstrates that PLK3 is frequently downregulated in tumor tissues compared to normal tissues. Low expression of PLK3 is correlated with poor survival of CRC patients. Moreover, we unravel a novel mechanism that PLK3 suppresses cells glucose metabolism via the HSP90/STAT3/HK2 pathway. PLK3 expression is directly suppressed by miR-106b in CRC cells. These findings point to a critical role of PLK3 in the colorectal cancer glycometabolism, which might provide a promising therapeutic target for the treatment of CRC.

## Supplementary information

**Additional file 1: Table S1.** Primer sequences used in this study.

**Additional file 2: Figure S1.** a PLK3 expression in eight CRC cell lines was tested by qPCR. b PLK3 expression in eight CRC cell lines was detected by immunoblotting. Densitometry represents the expression of the proteins relative to GAPDH. c SW480 and HCT116 cells transfected with pGLV-PLK3 and sh-PLK3, respectively, were subject to immunoblotting. Densitometry represents the expression of the proteins relative to GAPDH. d The relative mRNA expression of PLK1, PLK2, PLK4 and PLK5 in the cells with PLK3 silencing or overexpression.

**Additional file 3: Figure S2.** a Immunoblotting analysis verifying HK2 knockdown in SW480 and HCT116/sh-PLK3 cells. Densitometry represents the expression of the proteins relative to GAPDH. b Quantitative analysis of PLK3/HSP90 and HSP90/STAT3 interaction.

## Data Availability

The dataset supporting the conclusions of this article is included within the article.

## Abbreviations

ChIP: Chromatin immunoprecipitation
CRC: Colorectal cancer
HIF-1α: Hypoxia-inducible factor 1α
HK2: Hexokinase 2
HSP90: Heat shock protein 90
IHC: Immunohistochemistry
miRNAs: microRNAs
MZF1: Myeloid zinc finger 1
NF-κB: Nuclear factor κB
PLK: Polo-like kinase
qPCR: Quantitative real-time PCR
siRNAs: Small interfering RNAs
STAT3: Signal transducer and activator of transcription
