# Correction: Polo-like kinase 3 inhibits glucose metabolism in colorectal cancer by targeting HSP90/STAT3/HK2 signaling

**DOI:** 10.1186/s13046-026-03714-6

**Published:** 2026-04-21

**Authors:** Baochi Ou, Hongze Sun, Jingkun Zhao, Zhuoqing Xu, Yuan Liu, Hao Feng, Zhihai Peng

**Affiliations:** 1 Department of General Surgery, Shanghai General Hospital, Shanghai Jiao Tong University School of Medicine, No. 100, Haining Road, Shanghai, 200080 China; 2 Department of General Surgery, Ruijin Hospital, Shanghai Jiao Tong University School of Medicine, Shanghai, China

**Correction: J Exp Clin Cancer Res 38, 426 (2019)**

**https://doi.org/10.1186/s13046-019-1418-2**

Following publication of the original article [1], the authors found error in Fig. 2, specifically:

- Figure 2a - image depicting the effect of SW480 cancer cells transfected with vector plasmids on cells proliferation is incorrect.

Incorrect Fig. 2

**Fig. 2:**
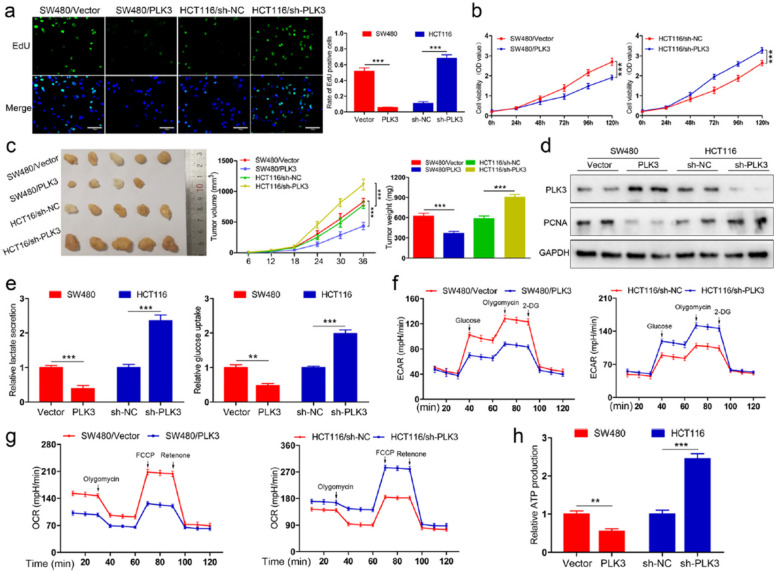
PLK3 suppresses proliferation and glucose metabolism of CRC cells. **a** Cell proliferation as measured by EdU assay was inhibited by PLK3 overexpression in SW480 cells and promoted by PLK3 knockdown in HCT116 cells. Scar bars, 200 μm. **b** CCK-8 assay showing PLK3 overexpression reduces cells viability of SW480 and PLK3 depletion enhances the viability of HCT116 cells. **c** Morphological observation of formed xenografts, the volumes measured every 6 days and average weight of tumors. **d** Immunoblotting analysis of PLK3 and PCNA in tumor samples. **e** Lactate production and glucose consumption of SW480 and HCT116 cells were determined. **f** Analysis of ECAR of PLK3-overexpressing SW480 cells and HCT116 cells with PLK3 knockdown. **g** Analysis of OCR of PLK3-overexpressing SW480 cells and HCT116 cells with PLK3 knockdown. **h** ATP production was examined in SW480 and HCT116 cells. Data represent the mean ± SD of at least three independent experiments. **P < 0.01, ***P < 0.001

Correct Fig. 2

**Fig. 2:**
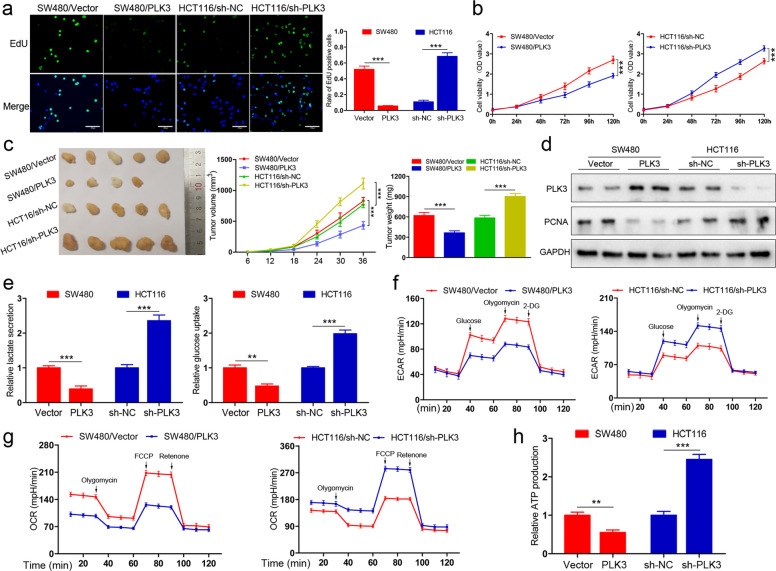
PLK3 suppresses proliferation and glucose metabolism of CRC cells. **a** Cell proliferation as measured by EdU assay was inhibited by PLK3 overexpression in SW480 cells and promoted by PLK3 knockdown in HCT116 cells. Scar bars, 200 μm. **b** CCK-8 assay showing PLK3 overexpression reduces cells viability of SW480 and PLK3 depletion enhances the viability of HCT116 cells. **c** Morphological observation of formed xenografts, the volumes measured every 6 days and average weight of tumors. **d** Immunoblotting analysis of PLK3 and PCNA in tumor samples. **e** Lactate production and glucose consumption of SW480 and HCT116 cells were determined. **f** Analysis of ECAR of PLK3-overexpressing SW480 cells and HCT116 cells with PLK3 knockdown. **g** Analysis of OCR of PLK3-overexpressing SW480 cells and HCT116 cells with PLK3 knockdown. **h** ATP production was examined in SW480 and HCT116 cells. Data represent the mean ± SD of at least three independent experiments. **P < 0.01, ***P < 0.001
