# Supplementary material for: Polo-like kinase 3 inhibits glucose metabolism in colorectal cancer by targeting HSP90/STAT3/HK2 signaling
Source: J Exp Clin Cancer Res. 2019 Oct 26;38:426. doi: 10.1186/s13046-019-1418-2 (PMC6815449; doi:10.1186/s13046-019-1418-2)
Supplement: Supplementary file 1 — Additional file 1: Table S1. Primer sequences used in this study. [file 13046_2019_1418_MOESM1_ESM.docx]

**Table S1** Primer sequences used in this study

| Primers used for qPCR: | | |
| --- | --- | --- |
| PLK3 forward | TTTTCGCACCACTTTGAGGAC | |
| PLK3 reverse | GAGGCCAGAAAGGATCTGCC | |
| PLK1 forward | CCTGCACCGAAACCGAGTTAT | |
| PLK1 reverse | CCGTCATATTCGACTTTGGTTGC | |
| PLK2 forward | CTACGCCGCAAAAATTATTCCTC | |
| PLK2 reverse | TCTTTGTCCTCGAAGTAGTGGT | |
| PLK4 forward | TTCTCGATACCTTCGTAGAGCTT | |
| PLK4 reverse | CTGAGTGACATCGTTCCATTGT | |
| PLK5 forward | CTCCCTGTCTGCGAAAGAGG | |
| PLK5 reverse | CCGTCCAAGAGCTGGTAGC | |
| HSP90 forward | GCTTGACCAATGACTGGGAAG | |
| HSP90 reverse | AGCTCCTCACAGTTATCCATGA | |
| GAPDH forward | CTGGGCTACACTGAGCACC | |
| GAPDH reverse | AAGTGGTCGTTGAGGGCAATG | |
| Primers for HK2 promoter construct: | Primer sequences | Enzyme |
| (-2131/-1505) HK2 sense: | TATAGTAGACAGAGAAATGGGAAAAGAT | AccI |
| (-2131/-1505) HK2 antisense: | GGTGTACATCAGGAGCAGAGGCACTGG | AauI |
| Primers used for ChIP in the HK2 promoter: |  | Position |
| STAT3 binding site sense: | GTCAGGCACCATTTTAGGCAC | -1980 bp |
| STAT3 binding site antisense: | TCAGGCTTTCCTTGACCAGT | -1800 bp |

**Table S2.** The expression level of HK2 in 116 pair of CRC specimens.

| Variable | Tissues (n=116) | | *P* value |
| --- | --- | --- | --- |
|  | Normal tissues | Carcinoma |  |
| HK2 expression |  |  | 0.001 |
| Negative | 64 | 38 |  |
| Positive | 52 | 78 |  |
