# Supplementary figures and images for: Polo-like kinase 3 inhibits glucose metabolism in colorectal cancer by targeting HSP90/STAT3/HK2 signaling
Source: J Exp Clin Cancer Res. 2019 Oct 26;38:426. doi: 10.1186/s13046-019-1418-2 (PMC6815449; doi:10.1186/s13046-019-1418-2)

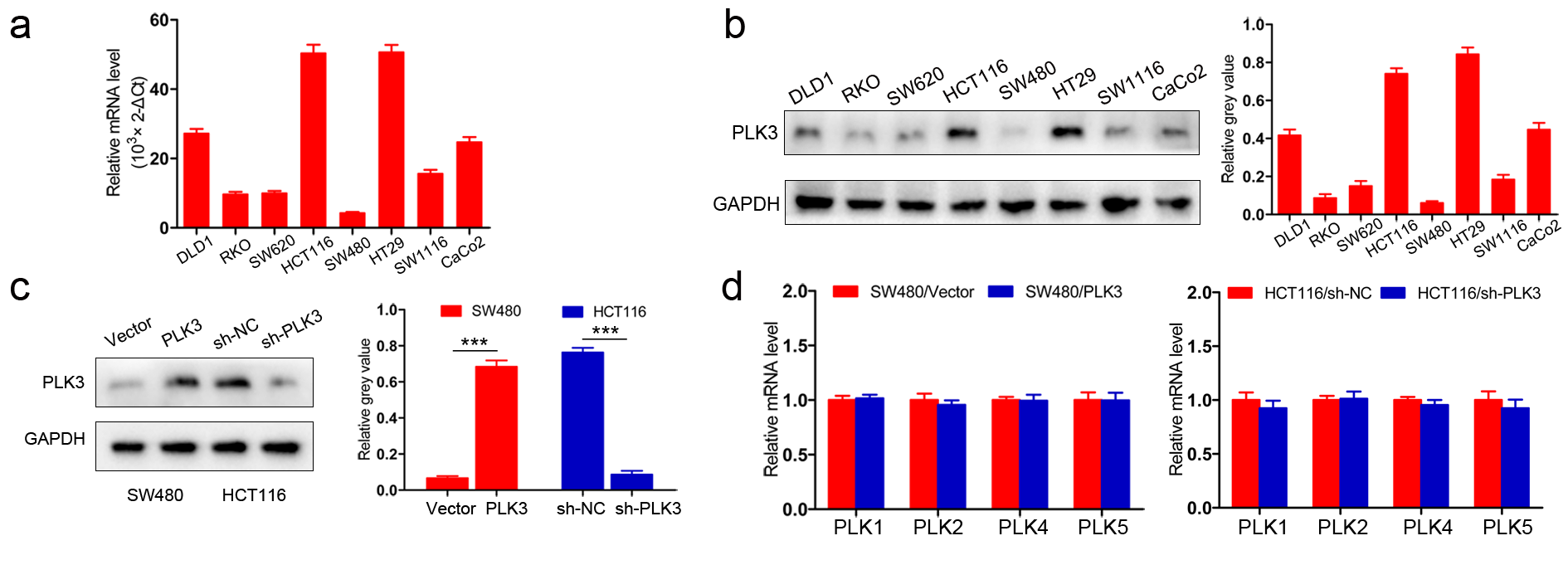

Supplement: Supplementary file 2 — Additional file 2: Figure S1. a PLK3 expression in eight CRC cell lines was tested by qPCR. b PLK3 expression in eight CRC cell lines was detected by immunoblotting. Densitometry represents the expression of the proteins relative to GAPDH. c SW480 and HCT116 cells transfected with pGLV-PLK3 and sh-PLK3, respectively, were subject to immunoblotting. Densitometry represents the expression of the proteins relative to GAPDH. d The relative mRNA expression of PLK1, PLK2, PLK4 and PLK5 in the cells with PLK3 silencing or overexpression. [file 13046_2019_1418_MOESM2_ESM.tif]

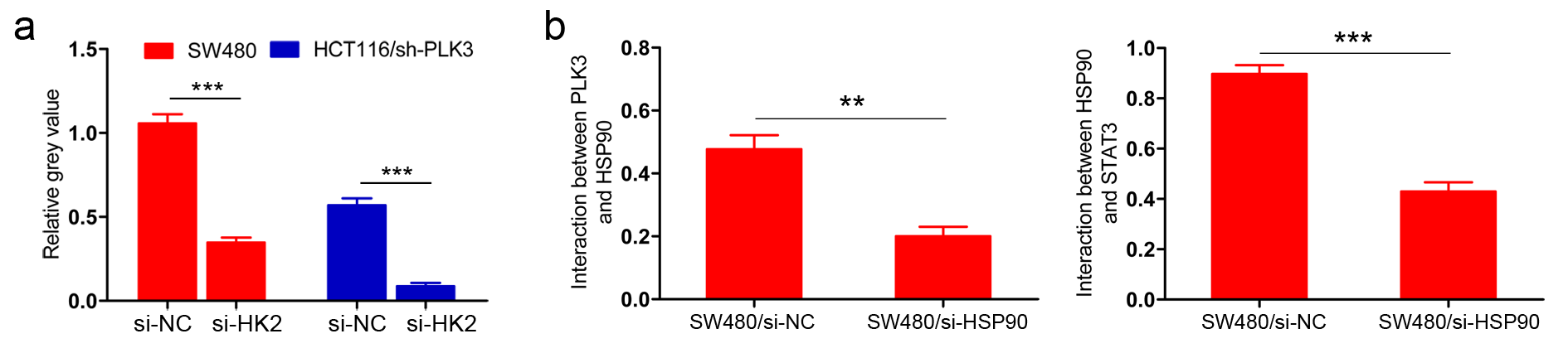

Supplement: Supplementary file 3 — Additional file 3: Figure S2. a Immunoblotting analysis verifying HK2 knockdown in SW480 and HCT116/sh-PLK3 cells. Densitometry represents the expression of the proteins relative to GAPDH. b Quantitative analysis of PLK3/HSP90 and HSP90/STAT3 interaction. [file 13046_2019_1418_MOESM3_ESM.tif]
